# Cheliensisin A (Chel A) induces apoptosis in human bladder cancer cells by promoting PHLPP2 protein degradation

**DOI:** 10.18632/oncotarget.11440

**Published:** 2016-08-20

**Authors:** Ruowen Zhang, Xun Che, Jingjie Zhang, Yang Li, Jingxia Li, Xu Deng, Junlan Zhu, Honglei Jin, Qinshi Zhao, Chuanshu Huang

**Affiliations:** ^1^ Nelson Institute of Environmental Medicine, New York University School of Medicine, Tuxedo, NY 10987, USA; ^2^ State Key Laboratory of Phytochemistry and Plant Resources in West China, Kunming Institute of Botany, Chinese Academy of Sciences, Kunming 650204, China; ^3^ Zhejiang Provincial Key Laboratory for Technology and Application of Model Organisms, School of Life Sciences, Wenzhou Medical University, Wenzhou, Zhejiang 325035, China

**Keywords:** Chel A, human bladder cancer, apoptosis, PHLPP2, c-Jun

## Abstract

Cheliensisin A (Chel A), a styryl-lactone compound extracted from *Goniothalamus cheliensis*, is reported to have significant anti-cancer effects in various cancer cells. Here we demonstrated that Chel A treatment resulted in apoptosis and an inhibition of anchorage-independent growth in human bladder cancer T24, T24T and U5637 cells. Mechanistic studies showed that such effect is mediated by PH domain and Leucine rich repeat Protein Phosphatases (PHLPP2) protein. Chel A treatment led to PHLPP2 degradation and subsequently increased in c-Jun phosphorylation. Moreover PHLPP2 degradation could be attenuated by inhibition of autophagy, which was mediated by Beclin 1. Collectively, we discover that Chel A treatment induces Beclin-dependent autophagy, consequently mediates PHLPP2 degradation and JNK/C-Jun phosphorylation and activation, further in turn contributing to apoptosis in human bladder cancer cells. Current studies provide a significant insight into understanding of anticancer effect of Chel A in treatment of human bladder cancer.

## INTRODUCTION

Cheliensisin A (Chel A, GC-51) has drawn a lot of interest in the last decade because of its potential role as an anti-cancer drug. Although previous studies reveal that Chel A has cytotoxicity against human leukemia cells *via* a PKA-dependent pathway by down-regulating Bcl-2 expression [1], the mechanism of Chel A's anti-cancer effects remains largely unknown. Our most recently studies have demonstrated that Chel A inhibits EGF-induced Cl41 cell transformation by stabilizing p53 protein [2]. In current study, we continued our efforts on Chel A's anti-cancer properties for apoptotic induction in human bladder cancer cells.

Bladder cancer is the fourth most common malignancy in men and the eighth most common in women in western world [3]. Bladder cancer is highly associated with environmental risk factors including smoking, poor nutrition and occupational exposure to various chemicals [4]. Because of such strong links to environmental risk factors, it is reasonable to use a natural chemical compounds to inhibit bladder cancer's development. Many naturally existing chemicals have been proven to interfere with the progression of bladder cancer to different extents [5, 6]. Here, we used the T24T cell line, a highly metastatic variant of the T24 bladder cancer cell line [7], to test the chemotherapeutic effects and mechanistic action of Chel A.

Many chemotherapeutic agents achieve their anti-cancer activity by inducing the apoptosis of cancer cells. C-Jun NH2-Terminal Kinase (JNK), a stress-activated protein kinase, is among the critical proteins involved in apoptosis upon various stress conditions [8]. It has been reported that the activation of JNK and its downstream transcription factor, c-Jun, is essential for apoptotic induction [9]. Our previous research also indicates that JNK is involved in apoptosis induced by resveratrol, a promising cancer preventive agent from grape extract [10]. We have also proved the involvement of the GADD45α-MKK4-JNK apoptotic cascade in the arsenite treatment [11]. Since JNK is only active in its phosphorylated state, dephosphorylation leads to its inactivation. In this study, we elucidated JNKs/c-Jun activation with the degradation of phosphatase PH domain and Leucine rich repeat Protein Phosphatases (PHLPP2), as well as apoptotic induction and anti-cancer effects in Chel A-treated human bladder cancer cells.

## RESULTS

### Chel A inhibited proliferation and anchorage-independent growth of bladder cancer cells

**Figure 1:**
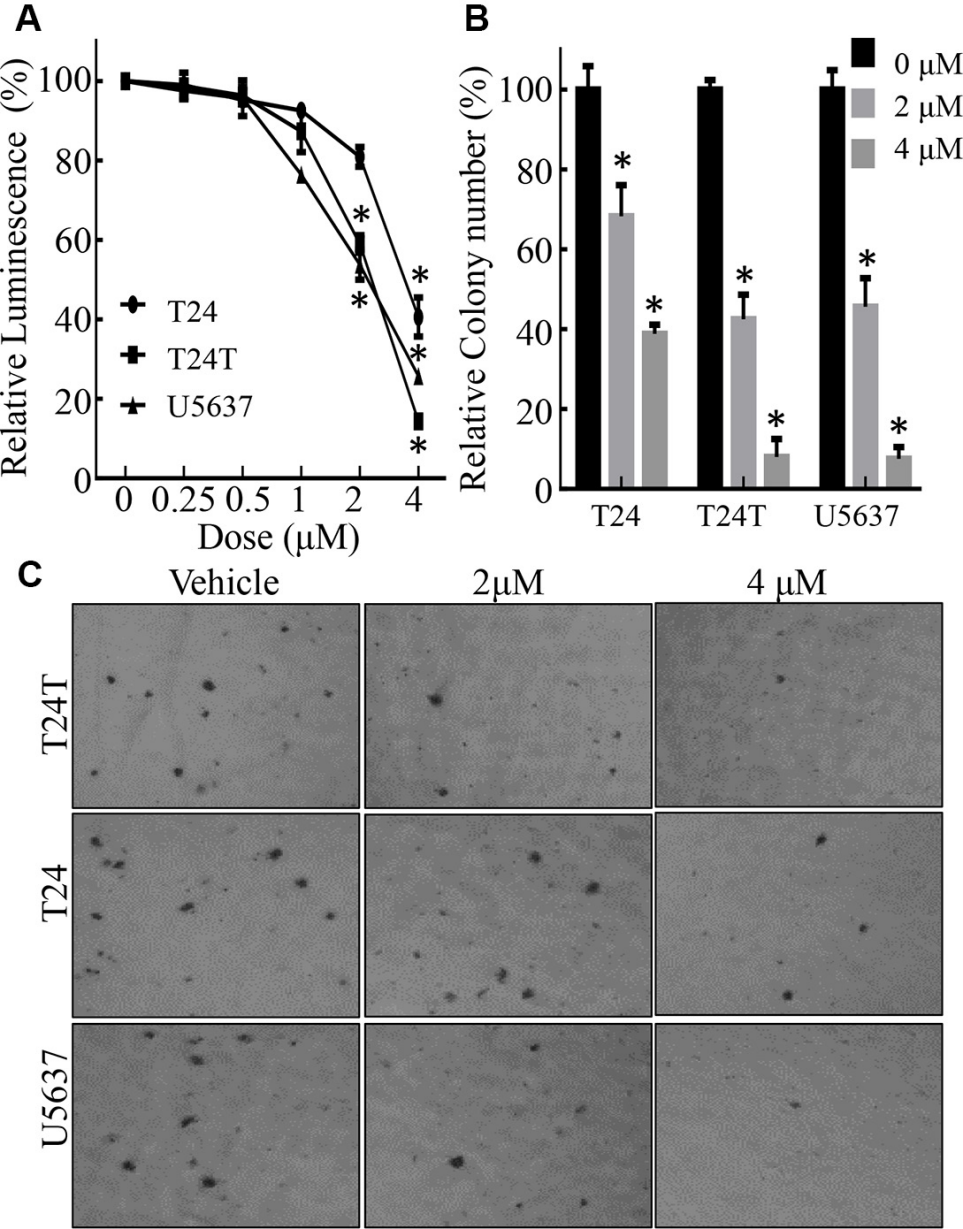
Chel A inhibits cell monolayer growth and anchorage-independent growth in bladder cancer cell lines (**A**) Results of a coupled ATPase activity assay in the presence of varying concentrations of Chel A at 24 hours. Incubation of the cells with Chel A resulted in dose-dependent growth retardation of T24, T24T and U5637 cells *in vitro* as observed in ATPase assays. Proliferation rates were determined in the indicated cells using a CellTiter-Glo Luminescent Cell Viability Assay kit. Results are presented as the mean ± S.D. of the triplicate assays. Error bars represent SD. (**B** and **C**) T24, T24T, U5637 cells were seeded in soft agar as described under “Materials and Methods”. Representative images of colonies in soft agar with or without Chel A were visualized under microscope and only colonies with over 32 cells were counted. Colonies are expressed as mean ± SD. from five assays of three independent experiments. The relative rate of inhibition is from the number of colonies from the Chel A-treated group, normalized by the number of colonies in the control group.

Chel A is a diterpenoid compound with a molecular weight of 648 kD [2]. To evaluate anti-cancer activities of Chel A in against human bladder cancer, three types of cell lines, T24, T24T and U5637, were treated with Chel A at different concentrations (0.25–4 μM) for 24 hours. Proliferation of these cells was analyzed using ATPase assay. As shown in Figure 1A, cell growth rate was significantly inhibited in all three tested bladder cancer cell lines in a dose-dependent manner. The IC50 of T24T, U5637, T24 cell lines, was 1.89 ± 0.02 μM, 1.93 ± 0.04 μM, 3.48 ± 0.64 μM, respectively. We then evaluated the potential inhibition of Chel A on an anchorage-independent growth of these three cells. The results showed that anchorage-independent growth of T24T, U5637 and T24 were dramatically attenuated by Chel A in a dose-dependent manner (Figure 1B and 1C), suggesting anti-cancer activity of Chel A in human bladder cancer.

### Chel A treatment induced apoptosis of human bladder cancer cells

**Figure 2:**
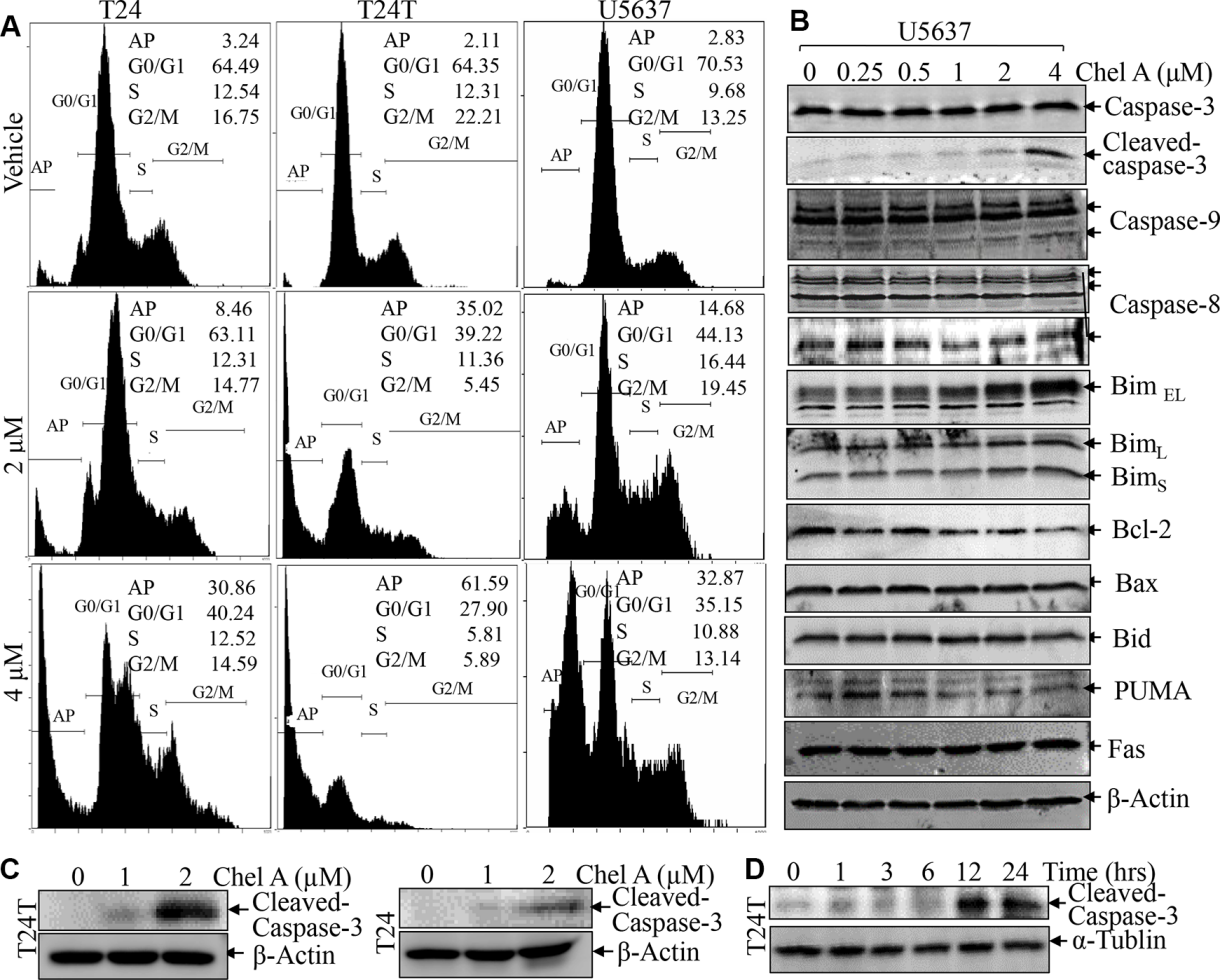
Chel A induces apoptosis in bladder cancer cell lines (**A**) After synchronization, the T24, T24T and U5637 cells were treated with Chel A as indicated for 24 hrs, and the cells were then fixed and subjected to cell cycle analysis by flow cytometry as described in “Materials and Methods”. The results represent one of three independent experiments. (**B** and **C**) After synchronization, the cells were treated with Chel A at the indicated concentrations for 24 hours or (**D**) Chel A at 4 μM for the time points indicated. The cell extracts were subjected to Western Blot with the indicated antibodies.

To elucidate the molecular mechanisms underlying Chel A anti-cancer effects, the potential effect of Chel A on induction of cell death and cell cycle was determined in bladder cancer cell lines, T24, T24T and U5637. These cell lines were exposed to 0, 2 or 4 μM Chel A for 24 hours and their effects on cell death and cell cycle were evaluated by flow cytometry. As shown in Figure 2A, a substantial increase in sub-G1 DNA content (apoptotic peak) was observed in all three cells treated with Chel A for 24 h. Since caspase3-cleavage is one of the hallmarks of apoptosis, we used its presence as a criterion to determine whether sub-G1 peak induced by Chel A was apoptotic cells. The results showed that Chel A treatment resulted in a dose-dependent increase in the cleavage of caspase3 proteins (Figure 2B and 2C). In addition, the Chel A-induced cleaved caspase-3 was observed in time-dependent manner (Figure 2D). These results suggest that Chel A treatment indeed induces human bladder cancer cell apoptosis, which might contribute to its anti-cancer activity in human bladder cancer cells.

### C-Jun phosphorylation was crucial for apoptotic induction following Chel A treatment

**Figure 3:**
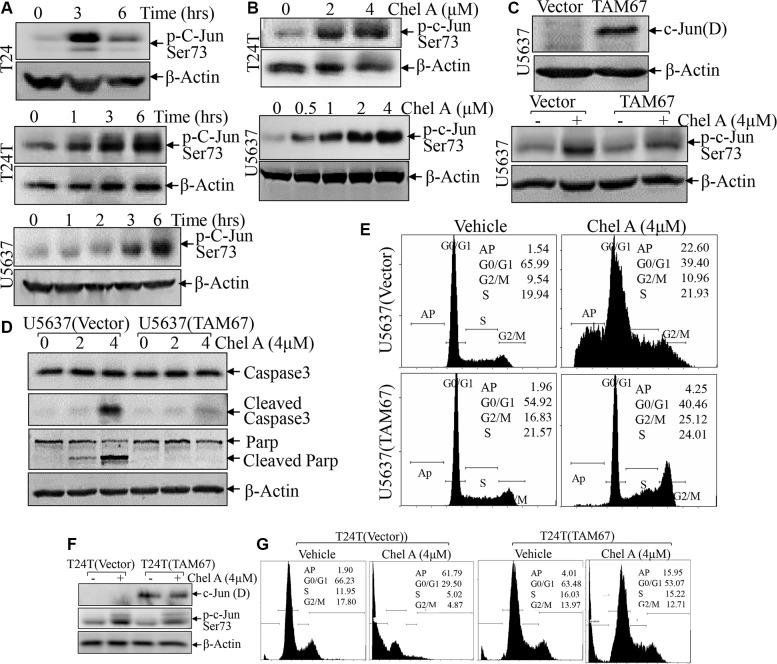
c-Jun phosphorylation up-regulated by Chel A plays a key role in the induction of bladder cancer cell apoptosis (**A** and **B**), After synchronization, T24 or T24T cells were treated with Chel A at 4 μM for at the time points indicated (A) or Chel A at the indicated concentrations for 6 hours (B). (**C** and **F**) T24T and U5637 cells stably transfected with TAM67 plasmids were seeded into 6-well plates. After synchronization, the cells were treated with or without 4 μM Chel A for 6 hrs. (**D**) U5637 cells stably transfected with TAM67 plasmids were seeded into each well of 6-well plates. After synchronization, the cells were treated with or without 4 μM Chel A for time indicated. The cell extracts were subjected to Western Blotting with the indicated antibodies. (**E** and **G**) The stable transfectants, T24(Vector) vs. T24T(TAM67) or U5637(Vector) vs. U5637(TAM67) cells, were treated with Chel A as indicated for 24 hrs, and the cells were then fixed and subjected to cell cycle analysis by flow cytometry. The results represent one of three independent experiments.

We next evaluated the effect of Chel A treatment on major regulators of cell apoptosis in U5637 cells. The results showed that Chel A treatment did not affect the expression of BCL-2 family proteins including Bcl-2, Bim, Bid, Bax and PUMA in U5637 cells (Figure 2B), whereas it induced a significant increase in c-Jun phosphorylation at Ser63 in all three cell lines (Figure 3A and 3B). To determine the role of phosphorylated-c-Jun in apoptotic induction by Chel A, TAM67, a c-Jun dominant negative mutant, was stably transfected into U5637 cells and the stable transfectants were treated with Chel A for apoptotic induction as compared with that in U5637(vector) control transfectants under same experimental conditions. As shown in Figure 3C and 3F, ectopic expression of TAM67 inhibited the c-Jun phosphorylation at Ser63 induced by Chel A. As expected, Chel A treatment resulted in a large proportion of sub-G1 peak in U5637 (vector) cells, while such induction was significantly blocked in U5637(TAM67) cells (Figure 3D). Consistently, the cleaved Caspase 3 and cleaved Parp levels were also significantly attenuated in U5637(TAM67) cells in comparison to those observed in U5637(vector) cells (Figure 3D). The apoptotic peak induced by Chel A was also impaired in U5637(TAM67) cells (Figure 3E). Similar results were also observed in T24T(TAM67) vs. T24T(vector) cells (Figure 3F and 3G). These results strongly demonstrate that C-Jun phosphorylation and activation was crucial for apoptotic induction in human bladder cancer cells following Chel A treatment.

### Down-regulation of PHLPP2 mediated Chel A-induced c-Jun phosphorylation, apoptosis and inhibition of anchorage-independent growth in human bladder cancer cells

**Figure 4:**
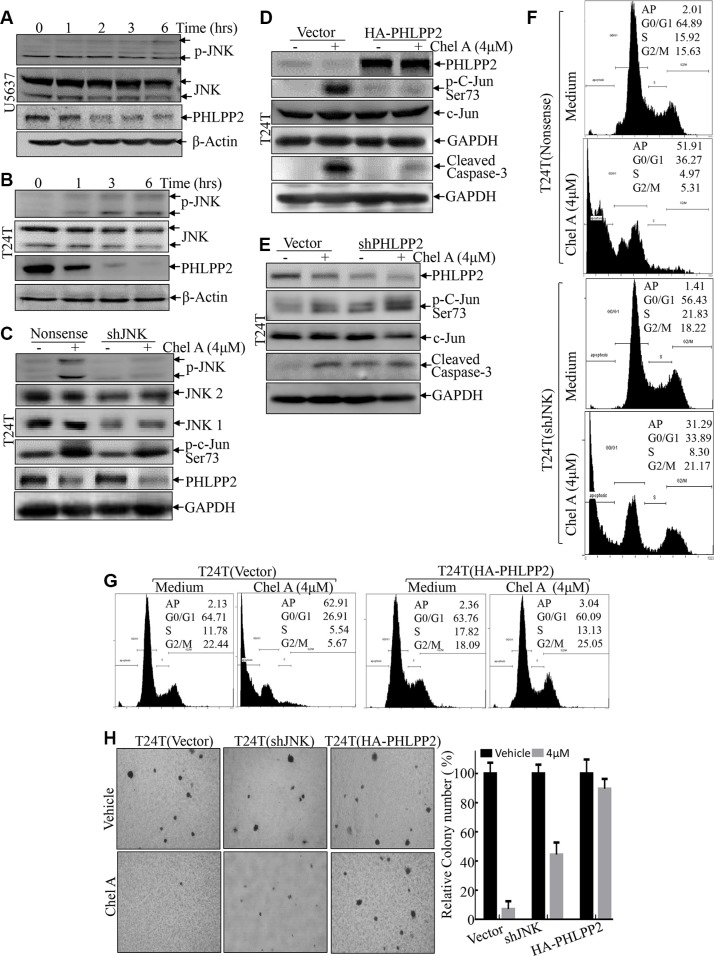
PHLPP2 mediates JNK and c-Jun phosphorylation (**A** and **B**) After synchronization, T24T and U5637 cells were treated with Chel A at 4 μM for the time points indicated. (**C** and **D**) T24T cells that were stably transfected with sh-JNK1&2, or HA-PHLPP2 plasmids were seeded into the wells of 6-well plates. After synchronization, the cells were treated with or without 4 μM Chel A for 6 hrs. The cells were then extracted and the cell extracts were subjected to Western Blotting with the indicated antibodies. (**E**) T24T cells that were stably transfected with shPHLPP2 plasmids or its control vector were seeded into the wells of 6-well plates. After synchronization, the cells were treated with or without 4 μM Chel A for 6 hrs. The cells were then extracted and the cell extracts were subjected to Western Blotting with the indicated antibodies. (**F** and **G**) The stable transfectants T24(vector) and T24T(sh-JNK1&2), T24T(vector) and T24T (HA-PHLPP2) cells were treated with Chel A as indicated for 24 hrs, and the cells were then fixed and subjected to cell cycle analysis by flow cytometry. The results represent one of three independent experiments. (**H**) T24(vector), T24T(sh-JNK1&2) and T24T(HA-PHLPP2) cells were seeded in soft agar as described under “Materials and Methods”. Representative images of colonies of these three cells in soft agar assay without or with Chel A at 4 μM were visualized under microscope and only colonies with over 32 cells were counted. Colonies are expressed as mean ± SD. from five assays of three independent experiments. The relative rate of inhibition is from the number of colonies from the Chel A treated group, normalized by the number of colonies in the control group.

The above results reveal that c-Jun phosphorylation and activation is a significant process responsible for apoptotic induction by Chel A. JNK is a well-known kinase for induction of c-Jun phosphorylation and activation [12]. Thus, JNK phosphorylation and activation was first evaluated in Chel A-treated cells. As shown in Figure 4A and 4B, following Chel A treatment, JNK phosphorylation was slightly observed in T24T and U5637 cells followed 3 hours of Chel A treatment, which is distinct from a marked increase in c-Jun phosphorylation. Such difference suggests that marked induction of c-Jun phosphorylation followed Chel A treatment might be regulated by either phosphatases or other kinases. This notion was greatly supported by results that the c-Jun phosphorylation at Ser63 was only partially alleviated in JNK shRNA stable transfectants, even though knockdown of JNK was specific and efficient (Figure 4C). Consistently, the results obtained from flow cytometry also showed that the only small proportion of cell apoptosis induced by Chel A decreased from 51.91% to 31.29% in T24T(sh-JNK) transfectants as compared to that from T24T(nonsense) transfectants (Figure 4F). Knockdown of JNKs only restored a small portion of anchorage-independent growth inhibited by Chel A treatment (Figure 4H). These results indicate that knockdown of JNKs only partially limits the effect of Chel A on apoptosis and anti-cancer activity. Meanwhile, markedly PHLPP2 downregulation was found as early as 2 hours after Chel A treatment in U5637 and T24T cell lines (Figure 4A and 4B). To investigate the correlation between the downregulation of PHLPP2 and c-Jun phosphorylation, the stable transfectant T24T (HA-PHLPP2) was established (Figure 4D). In contrast to resultant in downregulation of endogenous PHLPP2 expression, Chel A treatment did not affect exogenous HA-PHLPP2 expression (Figure 4D). Importantly, the c-Jun phosphorylation at Ser63 was impaired when PHLPP2 was overexpressed in T24T cells (Figure 4D). Conversely, knock-down of PHLPP2 in T24T cells increased in phosphorylation of C-Jun as well as cell apoptosis as indicated by caspase cleavage, even before the exposure to Chel A (Figure 4E). These results indicate the essential role of PHLPP2 in the phosphorylation and activation of c-Jun. To further evaluate how PHLPP2 down-regulation mediated the Chel A anti-cancer activity, we checked the effect of PHLPP2 overexpression on Chel A-induced cancer cell apoptosis and anchorage-independent growth. The results showed that cell apoptosis induced by Chel A could be blocked by introducing HA-PHLPP2 to T24T cells (Figure 4D and 4G). Along the same lines, Chel A's inhibition of T24T anchorage-independent growth was also completely reversed by the introduction of HA-PHLPP2 into T24T cells (Figure 4H). Collectively, our results demonstrate that Chel A-induced PHLPP2 downregulation plays a crucial role in the induction of c-Jun phosphorylation and apoptosis, as well as inhibition of T24T cell anchorage-independent growth, further revealing that PHLPP2 downregulation mediates anti-cancer activity of Chel A.

### Chel A downregulated PHLPP2 expression by promoting its protein degradation

**Figure 5:**
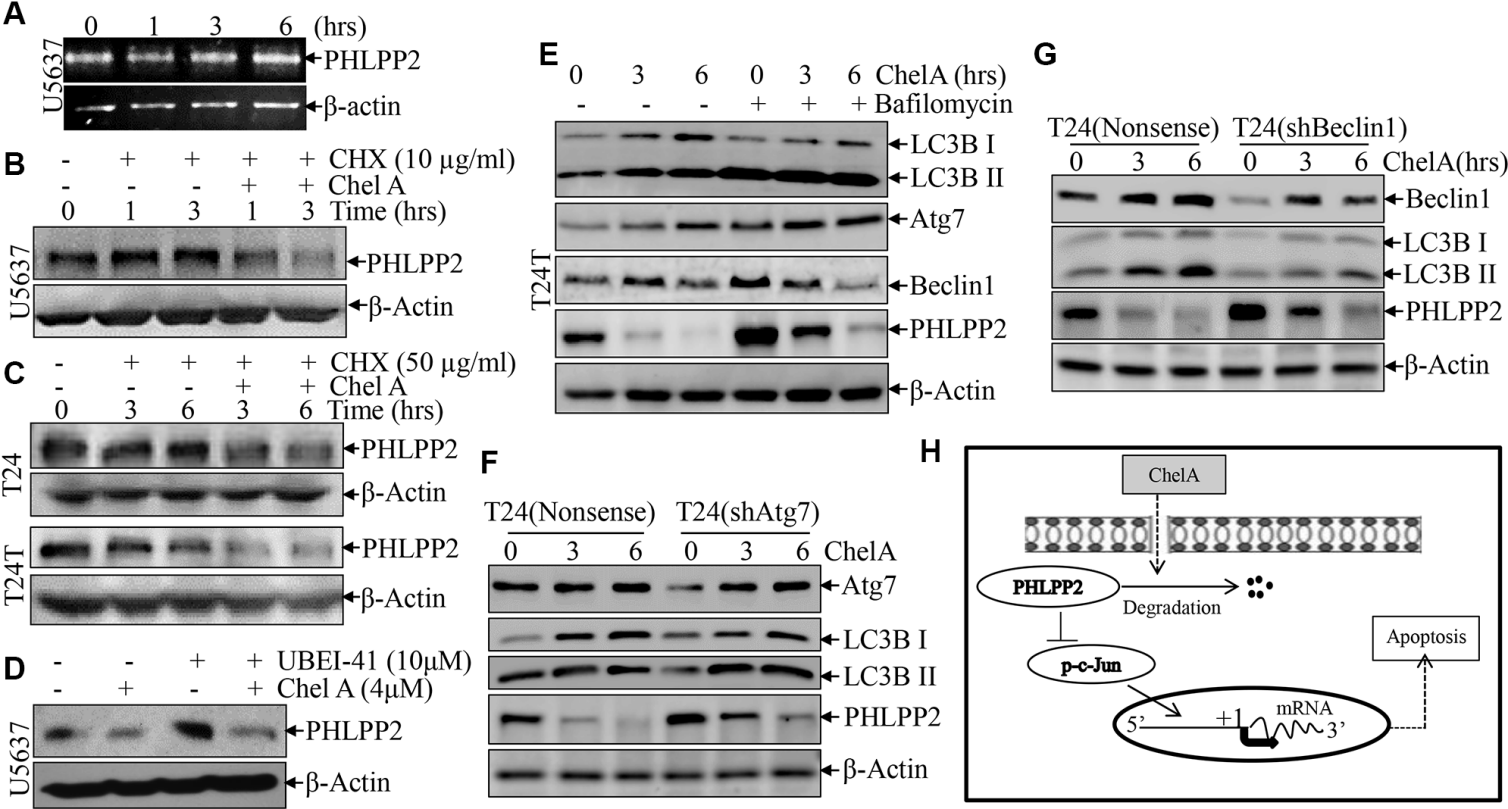
The down-regulation of PHLPP2 by Chel A is due to an increase in the rate of PHLPP2 protein degradation (**A**) Total RNA was isolated and subjected to RT-PCR analysis in the U5637 cells treated with 4 μM Chel A for the indicated time. PHLPP2 mRNA levels were evaluated by RT-PCR, with β-Actin mRNA levels used as loading control; (**B**) After synchronization, U5637 cells were pre-treated with MG132 for two hours. After MG132 was removed, cells were treated with or without 50 μg/ml CHX and/or 4 μM Chel A for time indicated, and the extracts were subjected to Western Blotting with anti-PHLPP2 or anti-β-Actin antibodies; (**C**) After synchronization, T24 or T24T cells were or were not exposed to 50 μg/ml CHX with or without 4 μM Chel A for the indicated time points, and the extracts were subjected to Western Blotting with anti-PHLPP2 or anti-β-Actin antibodies; (**D**) After synchronization, U5637 cells were respectively treated with or without 10 μM of UBEI-41 and with or without 4 μM of Chel A for three hours. The extracts were subjected to Western Blotting with anti-PHLPP2 or anti-β-Actin antibodies; (**E**) T24T cells were or were not incubated in 10 nM Bafilomycin A1 for 6 hours and treated with or without 4 μM Chel A. Cells were collected after time indicated and subjected to Western Blotting with anti-PHLPP2 or anti-β-Actin antibodies; (**F**) T24(shAtg7) and T24(Nonsense) were seeded into 6-well plates. After synchronization, the cells were treated with 4 μM Chel A for time indicated. The cell extracts were subjected to Western Blotting with the indicated antibodies. (**G**) T24(shBeclin1) and T24(Nonsense) were seeded into 6-well plates. After synchronization, the cells were treated with 4 μM Chel A for time indicated. The cell extracts were subjected to Western Blotting with the indicated antibodies. (**H**) The proposed model for the apoptotic responses following Chel A treatment.

The above results showed that the PHLPP2 downregulation plays a critical role in the increase of c-Jun phosphorylation as well as apoptosis and anti-cancer effect of Chel A compound. To elucidate the mechanisms underlying PHLPP2 protein downregulation upon Chel A treatment, the effect of Chel A treatment on PHLPP2 mRNA expression was evaluated. The results showed that Chel A treatment did not have any inhibitory effect on PHLPP2 mRNA levels for any of the time points tested (Figure 5A), suggesting that downregulation of PHLPP2 expression by Chel A could occur at either protein translation and/or degradation. The miRNAs have been reported to be involved in the regulation of protein translation by targeting 3′ UTR region of its regulated mRNA [13]. We, therefore, screened the potential miRNA binding sites on the 3′ UTR region of PHLPP2 mRNA from the database provided by miRBase (http://www.mirbase.org/search.shtml). The results indicated that the 3′UTR of PHLPP2 mRNA has a potential binding site for miR-31 (Figure S1A). To investigate whether miR-31 was a factor in the mediation of PHLPP2 protein expression, we first determined the miR-31 expression in U5637 cells treated with Chel A. As shown in Figure S1B in the Supplementary Data, the miR-31 expression mildly increased by 1.5-2 folds upon Chel A treatment. Then, we transfected synthetic miRNA expression constructs into U5637 cells and the results showed that ectopic expression of miR-31 did not show the any observable effect on expression of either PHLPP2 protein or its substrate Akt phosphorylation (Figure S1C). We finally focused on the effect of Chel A treatment on rate of PHLPP2 protein degradation. Interestingly, we observed that the treatment of U5637 cells with CHX for 1 or 3 hours did not cause PHLPP2 protein degradation, while treatment of cells with CHX together with Chel A for the same duration markedly promoted PHLPP2 protein degradation (Figure 5B). Consistently, the similar results were observed in both T24 and T24T cells (Figure 5C). Our results strongly indicate that Chel A inhibits PHLPP2 expression by the promoting PHLPP2 protein degradation. We next used UBEI-41, a cell-permeable ubiquitin E1 inhibitor that interrupts 26S proteasome-dependent protein degradation, to test whether Chel A-induced PHLPP2 protein degradation is *via* 26S proteasome-dependent manner. The results showed that UBEI-41 could not prevent the PHLPP2 from the degradation induced by Chel A although UBEI-41 was able to accumulate abundant PHLPP2 in U5637 cells (Figure 5D), suggesting that Chel A-induced PHLPP2 protein degradation is through 26S proteasome-independent manner. Autophagy is the basic catabolic mechanism regulating degradation of unnecessary or dysfunctional cellular components, which delivers cytoplasmic organelles to lysosomes for degradation [9], thus we then tested the possibility of PHLPP2 degradation *via* intracellular lysosomal autophagic pathway. The results showed that Chel A induced autophagic response in T24T cells as indicated by LC-3BII levels. Inhibition of Chel A-induced autophagy by application of Bafilomycin A1, an autophagy inhibitor, led to a markedly accumulation of PHLPP2 protein levels, as compared to that observed in T24T cells treated with Chel A alone (Figure 5E), revealing that PHLPP2 degradation might be involved in autophagy in human bladder cancer cells. It was noted that Chel A treatment led to increases in protein levels of two autophagy related proteins Atg7 and Beclin1 in T24T cells (Figure 5E). To test their potential participation in Chel A-induced autophagic responses and PHLPP2 protein degradation, we knocked down Atg7 and Beclin1 in T24 cells. We found that autophagy and PHLPP2 abundance upon Chel A treatment was only slightly affected by knockdown of Atg7 in T24 cells (Figure 5F), whereas knockdown of Beclin1 led to dramatically inhibition of autophagy accompanied with remarkably increased PHLPP2 protein abundance following Chel A treatment (Figure 5G). These results indicate that Chel A treatment leads to autophagy-mediated PHLPP2 protein degradation, and in turn results in JNK/C-Jun phosphorylation and activation, consequently leading to apoptosis of human bladder cancer cells, as briefly summarized in Figure 5H.

## DISCUSSION

Induction of apoptosis in pre-malignant cells is one of major mechanisms that have been responsible for chemotherapeutic effects of many anti-cancer agents. Chel A, being a natural compounds isolated from plant extracts, has been proven to have an inhibitive effect on leukemia by inducing apoptosis [1]. The very limitation of the knowledge on its anti-cancer effect partly limited the further usage of Chel A. To elucidate the molecular mechanism of Chel A, our present study demonstrated that Chel A treatment resulted in cell apoptosis and the inhibition of anchorage-independent growth of human bladder cancer cell lines, including T24, T24T and U5637 cells. Further studies showed that Chel A-induced c-Jun phosphorylation mediated apoptosis following Chel A treatment in T24, T24T and U5637 cells and that the rapid phosphorylation of c-Jun induced by Chel A was attributed to PHLPP2 downregulation. Moreover, we found that PHLPP2 downregulation upon Chel A treatment was due to its promoting PHLPP2 protein degradation, which resulted from autophagy induction by Chel A treatment. Therefore, we identify the novel molecular mechanisms underlying the anti-cancer activity of Chel A compound by targeting PHLPP2/JNK-c-Jun apoptotic axis.

The activation of c-Jun exhibits multiple effects on the regulation of cell growth and oncogenic transformation [14, 15], as well as mediating cell death either by acting as a transcriptional regulator or by triggering caspase-mediated cleavage of proteins [16, 17]. Potential explanations for its opposing effects on cell death include cell-type specificity, the availability of external and internal survival factors, as well as specific spectra of binding partners [18]. Recently, it was reported that the duration of c-Jun activation is a critical factor in determining whether the cell survives or undergoes apoptosis [19]. In this study, Chel A-induced apoptosis was accompanied by the phosphorylation and activation of c-Jun as early as an hour after Chel A treatment and lasted for at least 24 hours; inhibition of the phosphorylation of c-Jun resulted in a significant reduction in cell apoptosis. It is important to note that interference with JNK activation did not result in the total abolition of Chel A-induced cell death, as shown by flow cytometry assay. This may be due to an incomplete inhibition of the c-Jun phosphorylation and activation in JNK knockdown transfectants. Furthermore, c-Jun was activated as early as an hour after Chel A treatment, while the activation of JNK appeared more than two hours later. These results indicate that an alternative upstream regulator being mediating c-Jun phosphorylation is not through c-Jun kinase JNKs.

PHLPP represents a family of novel Ser/Thr protein phosphatases [20]. There are two isoforms of PHLPP, PHLPP1 and PHLPP2, which negatively regulate Akt serine-threonine kinases (Akt1, Akt2, Akt3) and protein kinase C (PKC) isoform [21]. Since PHLPP is able to dephosphorylate Akt at Ser-473 and block growth factor-induced signaling in cancer cells [22], it plays a critical role in tumor suppression in several types of cancer. Unfortunately, the genetic loci coding for PHLPP1 and 2 are commonly lost in cancer, and expression of their isoform is also lost or decreased in some tumor tissues. Experimental overexpression of PHLPP in these cancer cell lines tends to induce apoptosis and attenuates proliferation of cancer cells. In contrast, it is interesting to note that the overexpression of PHLPP2 in T24T cells is effective in blocking the anti-cancer activity of Chel A. This strongly demonstrates that PHLPP2 not only has the effect of blocking growth factor-induced signaling in cancer cells as reported before, but it also serves the novel function as a target of anti-cancer reagents. As we mentioned, JNK is not a major kinase being responsible for phosphorylating c-Jun. These results indicate that Chel A treatment causes a down-regulation of PHLPP2 expression, which is the critical factor for mediating phosphorylation and activation of c-Jun, where JNK only plays a minor part. We also found that PHLPP2 exhibited dephosphorylation of c-Jun *via* direct binding [21, 23]. Following this information, we tried to find out how Chel A downregulates PHLPP2 protein. Since PHLPP2 protein was downregulated as early as an hour after Chel A treatment, we anticipated that Chel A down-regulated PHLPP2 by enhancing protein degradation, which was proved by the studies demonstrated in our current studies. However, it was surprising that inhibition of E1 enzyme by UBEI-41 could not prevent PHLPP2 protein degradation, indicating that the protein degradation induced by Chel A might be ascribed to the protein degradation in ubiquitination-independent mechanism. Thus, we further tested possible association of Chel A-induced PHLPP2 degradation with the lysosomal-dependent degradation. The results have revealed that autophagy induced by Chel A contributed to PHLPP2 protein degradation. With utilization of shRNA knockdown approach, our studies have indicated that Beclin1, but not Atg7, was the mediator of autophagic responses and in turn leading to PHLPP2 protein degradation, although the detailed the mechanisms are still under exploration in our laboratory.

In conclusion, our results demonstrate that Chel A's anti-cancer activities are achieved by down-regulating PHLPP2 *via* protein degradation, subsequently increasing the phosphorylation of c-Jun accumulation, and cancer cell apoptosis, as well as anchorage-independent growth retardation as diagramed in Figure 5H. These results provide important new insights into the understanding of the molecular basis underlying the nature of Chel A anti-cancer activity, further contributing to the design and synthesis of other new conformation-constrained derivatives for the treatment of cancers. Furthermore, our study discloses a novel function of PHLPP2: dephosphorylating c-Jun to promote bladder cancer cell growth and to induce its apoptosis.

## MATERIALS AND METHODS

### Chemicals and antibodies

Chel A (6-(7,8-epoxy-styryl)-5-acetoxy-5,6-dihydro-2-pyrone; Figure 1A) was isolated from *Goniothalamus cheliensis* by the Kunming Institute of Botany, Chinese Academy of Sciences (Kunming, China) in the form of a white crystal with a purity of more than 99.0%, as previously described [2]. Chel A was dissolved in Dimethyl sulfoxide (DMSO) to make a stock concentration at 80 mM and further diluted in DMEM with final DMSO concentration at 0.1% (v/v) for cell treatment. DMSO of the same concentration (0.1%, v/v) was used as a vehicle control in all experiments. The chemicals cycloheximide (CHX) was purchased from Calbiochem (San Diego, CA, USA). Bafilomycin A1 was purchased from Santa Cruz Biotechnology (Dallas, TX, USA). Antibodies specific against cleaved-caspase3, caspase3, caspase8, caspase9, Bim, Bcl-2, Bax, Bid, LC3B, PUMA, Fas, phospho-p38 (Thr180/Tyr182), phosphor-JNK (Thr183/Tyr185), JNK, JNK1, c-Jun, phosphor-c-Jun(Ser73), GAPDH, α-Tubulin, β-Actin were purchased from Cell Signaling (Danvers, MA, USA). Antibody specific against PHLPP2 was purchased from Bethyl Laboratories (Montgomery, TX, USA). Anti-HA antibody was purchased from Covance Antibody Service Inc. (Princeton, NJ, USA).

### Cell lines and culture

The Human T24T bladder cancer cell line was a gift from Dr. Dan Theodorescu [24] and used in our previous studies [13]. Human T24 bladder and U5637 cancer cells were a gift from Dr. Xue-Ru Wu, Departments of Urology and Pathology, New York University School of Medicine. These cell lines were maintained in 1:1 mixture of Dulbecco's Modified Eagle's Medium (DMEM)/Ham's F-12 medium and Dulbecco's Modified Eagle's Medium (DMEM), supplemented with 5% (v/v) or 10% (v/v) heat-inactivated FBS, 2 μM L-glutamine, and 25 μg/ml gentamycin, respectively, at 37°C in a humidified atmosphere of 5% CO_2_. MEF (mouse embryonic fibroblast) cells were described in our previous study [13].

### Plasmids and transfection

TAM67 plasmid (dominant negative mutant c-Jun) was a kind gift from Drs. Nancy Colburn and Matthew Young from National Cancer Institute and Tim Bowden from the University of Arizona and has been used in our previous study [21]. The shRNA constructs targeting JNK1, JNK2, PHLPP2, Atg7 and Beclin1, were purchased from Open Biosystems (Pittsburgh, PA, USA). The HA-PHLPP2 plasmid was purchased from Addgene (Plasmid 22403) (Cambridge, MA, USA). T24T or and U5637 cells were transfected with the indicated constructs using PolyJetTM DNA *in vitro* transfection reagent (SignaGen Laboratories, Rockville, MD) according to manufacturer's instructions and the stable transfectants were established by selecting with hygromycin for 4–6 weeks, and surviving cells were pooled as stable mass transfectants as described in our previous studies [12].

### Cell proliferation assay

Confluent monolayers of cells were trypsinized, and 1 × 10^3^ viable cells suspended in 100 μL of normal cell culture medium were added to each well of a 96-well plate. After 12 hour incubation at 37°C in a humidified atmosphere of 5% CO_2_, cells were synchronized by culturing with 0.1% FBS medium for another 24 h. The cells were then cultured in 10% FBS medium for the time as indicated and the proliferative rate was determined using the CellTiter-Glo Luminescent Cell Viability Assay kit (Promega, Madison, WI) with a luminometer (Wallac 1420 Victor2 multipliable counter system) as described in our previous publications [13].

### Anchorage-independent growth assay in soft agar

Anchorage-independent growth assay in soft agar (soft agar assay) was carried out as described in our previous study [20]. Briefly, 1 × 10^4^ cells, in 10% FBS BME medium containing 0.33% soft agar, with or without 4 μM of Chel A, were seeded over bottom layer of 0.5% agar in 10% FBS BME medium in each well of 6-well plates. The plates were incubated in 5% CO_2_ at 37°C for 3 weeks. Colonies were observed under microscope and only colonies with over 32 cells were counted, the images were captured as well. The results were presented in colonies/10^4^ cells.

### Western blot

Western Blot was generally carried out as described in our previous study [22]. Briefly, cells were seeded in the 6-well plates and cultured until 70–80% confluent in 1:1 mixture of Dulbecco's Modified Eagle's Medium (DMEM)/Ham's F-12 medium supplemented with 5% (v/v) heat-inactivated FBS. The culture medium was replaced with 0.1% FBS media for 24 hours. The cells were then treated with Chel A as indicated in the figure legends, and the cells were extracted and cell extracts were subjected to Western Blot. The proteins recognized by specific primary antibodies were detected by an alkaline phosphatase-linked secondary antibody and an ECF Western Blot system (Amersham Biosciences, Piscataway, NJ).

### Reverse transcription polymerase chain reaction (RT-PCR)

Total RNA was extracted with TRIzol reagent (Invitrogen Corp., Carlsbad, CA), and cDNAs were synthesized with the Thermo-Script RT-PCR system (Invitrogen Corp., Carlsbad, CA). The human β-actin cDNA used as internal control was amplified using two specific primers 5′-GCG AGA AGA TGA CCC AGA TCA T-3′(sense) and 5′-GCT CAG GAG GAG CAA TGA TCT T-3′ (antisense). The human PHLPP2 cDNA fragments were amplified by Forward: 5′-AGG TTC CTG AGC ATC TCT TC-3′, Reverse: 5′-GTT CAG GCC CTT CAG TTG AG-3′. The RT-PCR products were analyzed on 2% agarose gels and the images were scanned and visualized after staining with ethidium bromide with FluorChem SP imaging system (Alpha Innotech Inc., CA, USA).

### Flow cytometry assay

The cell cycle distributions were determined by Flow cytometry as described previously [25]. The cells were cultured in 6-well plates till 60% confluent and the cell culture medium was replaced with 0.1% FBS medium and cultured for 24 hrs for synchronization. The cells were treated with Chel A for 24 hrs, and then collected with ice-cold PBS and fixed with 5 ml of 75% ethanol at −20°C overnight. The fixed cells were stained in the buffer containing 0.1% Triton X-100, 0.2 mg/ml RNase A, and 50 μg/ml PI (propidium iodide) and were then subjected to EpicsXL flow cytometer (Beckman Coulter Inc., Miami, FL) for cell cycle analysis. The results were analyzed by histogram analyses software (Expo32 v1.2).

### Real-time PCR

Total RNA was extracted with TRIzol reagent (Invitrogen Corp., Carlsbad, CA), and cDNAs were synthesized with the Thermo-Script RT-PCR system (Invitrogen Corp., Carlsbad, CA). Real time PCR was used to measure the expression level of miR-31 mRNA levels utilizing a previously described miR-31 specific primer AGG CAA GAT GCT GGC ATA GCT G, (Invitrogen Corp., Carlsbad, CA, USA). The level of miR-31 mRNA expression was normalized to that of β-actin assessed by the same assay with the primer sequences being sense, CGA CAA CGG CTC CGG CAT GT, and antisense, TGC CGT GCT CGA TGG GGT ACT. (Invitrogen Corp., Carlsbad, CA)

### Statistical methods

Student's *t* test was applied to data analysis and *P* < 0.05 was considered statistically significant.

## SUPPLEMENTARY MATERIAL FIGURE


