# Supplementary material for: Cheliensisin A (Chel A) induces apoptosis in human bladder cancer cells by promoting PHLPP2 protein degradation
Source: Oncotarget. 2016 Aug 20;7(41):66689–99. doi: 10.18632/oncotarget.11440 (PMC5341830; doi:10.18632/oncotarget.11440)
Supplement: Supplementary file 1 [file oncotarget-07-66689-s001.pdf]

# Cheliensisin A (Chel A) induces apoptosis in human bladder cancer cells by promoting PHLPP2 protein degradation

## Supplementary Materials

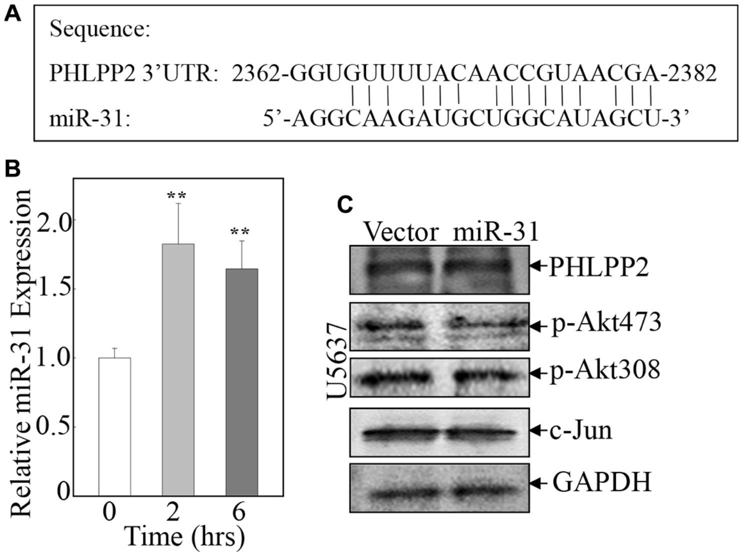


**Supplementary Figure S1:** (**A**) shows a sequence complementarity between PHLPP2 and miR-31. (**B**) After being exposed to 4 µM Chel A for the indicated time points, U5637 cells were harvested for RNA isolation. QT-PCR was employed to examine the relative expression of miR-31. (**C**) Stable overexpression of miR-31 transfectants were established in the U5637 cells. After synchronization, U5637 (vector) and U5637 (miR-31) were harvested for Western Blotting with the indicated antibodies.
